# Dietary Flaxseed as a Strategy for Improving Human Health

**DOI:** 10.3390/nu11051171

**Published:** 2019-05-25

**Authors:** Mihir Parikh, Thane G. Maddaford, J. Alejandro Austria, Michel Aliani, Thomas Netticadan, Grant N. Pierce

**Affiliations:** 1 Department of Physiology and Pathophysiology, College of Medicine, Faculty of Health Sciences, University of Manitoba, Winnipeg, MB R3E 0W3, Canada; mparikh@sbrc.ca (M.P.); tmaddaford@sbrc.ca (T.G.M.); aaustria@sbrc.ca (J.A.A.); tnetticadan@sbrc.ca (T.N.); 2 Canadian Centre for Agri-Food Research in Health and Medicine (CCARM), Albrechtsen Research Centre, St Boniface Hospital, 351 Taché Avenue, Winnipeg, MB R2H 2A6, Canada; maliani@sbrc.ca; 3 Institute of Cardiovascular Sciences, Albrechtsen Research Centre, St. Boniface Hospital, 351 Taché Avenue, Winnipeg, MB R2H 2A6, Canada; 4 Department of Human Nutritional Sciences, Faculty of Agricultural and Food Sciences, University of Manitoba, Winnipeg, MB R3T 2N2, Canada; 5 Morden Research and Development Centre, Agriculture and Agri-Food Canada, Morden, MB R6M 1Y5, Canada

**Keywords:** flaxseed, cancer, cardiovascular, microbiome, diabetes, menopause, nutraceutical, functional food

## Abstract

Flaxseed is a rich source of the omega-3 fatty acid, alpha linolenic acid, the lignan secoisolariciresinol diglucoside and fiber. These compounds provide bioactivity of value to the health of animals and humans through their anti-inflammatory action, anti-oxidative capacity and lipid modulating properties. The characteristics of ingesting flaxseed or its bioactive components are discussed in this article. The benefits of administering flaxseed or the individual bioactive components on health and disease are also discussed in this review. Specifically, the current evidence on the benefits or limitations of dietary flaxseed in a variety of cardiovascular diseases, cancer, gastro-intestinal health and brain development and function, as well as hormonal status in menopausal women, are comprehensive topics for discussion.

## 1. Introduction

Dietary flaxseed has an impressive and growing research literature supporting its use in a variety of health conditions. Whereas in the late 20th century, little was known of the health-related benefits of flaxseed as well as how to consume flaxseed to achieve any health benefits, today is an entirely different time. Research information on the effects of dietary flaxseed has increased dramatically. We now know what diseases flaxseed can treat or prevent, the health impacts dietary flaxseed can provide, the bioactives within flaxseed that provide these health-related effects in many cases and the forms of flaxseed that are required to provide these bioactives to the body. Data on the impact of dietary flaxseed on disease have been translated, albeit in limited amounts, from animal research studies to human trials. Many of these trials have been conducted with the highest standards of control available in order to provide solid, reliable information on its value to the general public and, more specifically, to those in a disease compromised condition. These data have sparked the food processing industry to create a greater variety of foods that contain flaxseed, giving the public an increased opportunity to incorporate flaxseed into their everyday diet. With this growing availability of flaxseed in the marketplace and the increasing awareness of the public to its impact on human health when supplemented into the diet on a daily basis, it would be expected that flaxseed will also grow in the coming years in economic importance to the farmer, the food processer and the retailer in the marketplace [1].

We now know that environmental factors like diet can have a profound effect on the maintenance of health and the appearance of disease [2]. It is also increasingly apparent that the treatment of disease is not only achieved through pharmacological therapy, but through dietary interventions as well. The purpose of this review is to provide a broad summary of the highlights of the research that have supported the growth of flaxseed as a commodity with significance in the fields of health and medicine.

## 2. Flaxseed and Its Use in the Diet

The main bioactive compounds in flaxseed include alpha-linolenic acid (ALA), lignans and fiber. Four common forms of flaxseed available for human consumption include whole flaxseed, ground flaxseed, flaxseed oil and partially defatted flaxseed meal [3]. A new form available in the marketplace is flax “milk” (Pizzey Ingredients Inc, Manitoba, Canada). An alternative to “milks” like almond milk, flax milk is finely milled flaxseed mixed with filtered water and other minor compounds. Flax milk is high in ALA and is an excellent alternative to dairy milk, as it has no cholesterol or lactose. It is suitable for people allergic to soy, nuts and gluten, and it contains more health benefits than almond milk. 

However, no matter how good a food is for the general public to consume, if that food does not have acceptable taste, texture, appearance, colour and aroma qualities, then the majority of people will not eat it. Flaxseed has several characteristics that could negatively affect its flavour profile and that are cause for concern. The two most important are the potential for the very high omega-3 fatty acid content in flaxseed to become rancid through oxidation, and the propensity for bitterness. The high content of alpha linolenic acid (ALA) renders it highly susceptible to oxidation. The oxidation and subsequent rancidity will lead to off flavours and a musty aroma that would be rejected in taste tests. The antioxidant content in flaxseed, provided in turn by its secoisolariciresinol diglucoside (SDG) content, is of great value in curtailing any oxidation process. In addition, flaxseed has been described as possessing a “nice nutty smell and aroma” [4], and is potentially ideal for the incorporation into a variety of foods. In research studies, flaxseed has been incorporated successfully into snack bars, muffins, bagels, bread, buns, tea biscuits, cinnamon rolls and pasta [5,6]. The concentration of flaxseed in the food will, of course, influence its flavour characteristics. Flaxseed has been incorporated into foods for human consumption at concentrations of 5–28% of total ingredients (by weight) before baking [7]. The amount of flaxseed ingested daily over an extended period of time has been as much as 40 to 50 g [8]. Clinical trials having patients ingesting a food each day for up to one year have been successfully completed with drop-out rates of approximately 20% in both placebo and flax groups [5]. This demonstrates that it is not the flaxseed that induces non-compliance *per se*, but instead a great number of different flax-containing foods are needed to allow variety in the daily choices if one is to maintain subjects in a trial with an extended duration. 

The most common foods in which to have flaxseed are baked goods. The process of baking even up to 178 ℃ for two hours does not alter the composition or content of ALA in a baked muffin, for example [9]. The addition of flavouring to the baked good also makes it possible to disguise any less than optimal flavour profile induced by the bitterness of flaxseed or any minor rancidity. Flavourings like banana and nuts, banana chocolate chip, cinnamon apple raisin, cinnamon raisin, cinnamon spice, gingerbread raisin, cappuccino chocolate chip, sunflower sesame and cranberry orange have been used in research studies [7,10]. The public will have preferences to these flavourings and matrices for the flaxseed. In a study of the daily preferences of the ingestion of food products containing 30 g of milled flaxseed over one year, bagels were consumed > muffins > snack bars > sprinkles > tea biscuits > pasta > sundried tomato buns [11]. Cinnamon raisin flavourings were preferred > cranberry orange > apple spiced > sunflower sesame [11]. The recent development of flax “milk” provides an even more convenient way to consume flaxseed and preserve it as well.

Each of these different food matrices can influence the stability of the flaxseed. Processing of the seed, storage temperature and duration as well as the form of flaxseed (milled flaxseed versus whole seed versus flax oil) will also influence the stability of the product. Milling, grinding or crushing flaxseed will destroy the hard protective seed coat on flaxseed, exposing the ALA and SDG to oxidation [12]. However, this process is required to render these bioactive ingredients bioavailable [13]. ALA is more bioavailable to the body if it is in oil or milled form [13]. The incorporation of milled flaxseed into baked products may actually protect the ALA and SDG from degradation [12]. Cooler storage temperatures and a shortened storage duration, particularly for flax oil, will also result in a better preservation of the ALA and SDG [12].

Recently, a concern was raised about the presence of components in flaxseed that may have undesirable effects that could affect the bioavailability and bioaccessibility of essential nutrients [14]. These include the presence of protease inhibitors, phytic acids, linatine and cyanogenic glycosides in flaxseed. However, there are no deleterious effects reported of these components in human studies. The concentrations of these components delivered through dietary flaxseed may be below that needed to induce any biological actions. Nevertheless, it is prudent to take into account the raised concern. Plant bio-breeding and/or food processing methods may be considered to reduce the levels of these components in flaxseed. 

## 3. Dietary Flaxseed and Cardiovascular Disease

**Figure 1:**
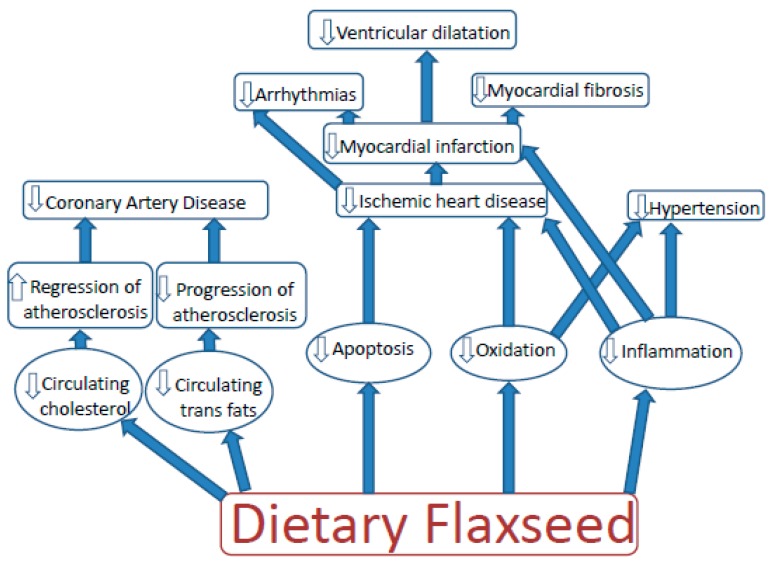
The effect of dietary flaxseed on the various types of cardiovascular disease. Arrows going up denote an increase in a specific parameter whereas arrows going down denote a decrease in a specific parameter.

The effects of flaxseed on parameters of cardiovascular disease have become one of the most intensely studied areas with regard to the health-related benefits of dietary flaxseed (Figure 1). 

In animal models of heart disease, dietary flaxseed has decreased the progression of atherosclerosis induced by high dietary cholesterol or high dietary trans fat content [15,16,17], likely via an anti-inflammatory action provided through its ALA content [15,16,18]. Depending upon the animal species, flaxseed may (mice and rats) or may not (rabbits) lower circulating cholesterol levels [15,16,18]. Flaxseed can also lower circulating trans fats levels [17]. In a stable, established atherosclerotic plaque, supplementation of the diet with flaxseed can also regress atherosclerosis [19]. 

Ischemic reperfusion challenge to isolated hearts can induce arrhythmias. Rabbits on a flaxseed supplemented diet prior to being subjected to the ischemic reperfusion insult are protected from ventricular fibrillation [20]. If rats are placed on a flaxseed supplemented diet prior to and after the induction of a myocardial infarction, they exhibit smaller infarcts, less arrhythmias and a reduction in left ventricular dilation [18]. The mechanism once again is likely to involve the ALA content of flaxseed [18]. ALA has shown a capacity to alter arrhythmogenic ionic currents in the heart [21]. However, the metabolism of SDG into enterolignans within the circulation may also have a cardioprotective role [18,22].

**Figure 2:**
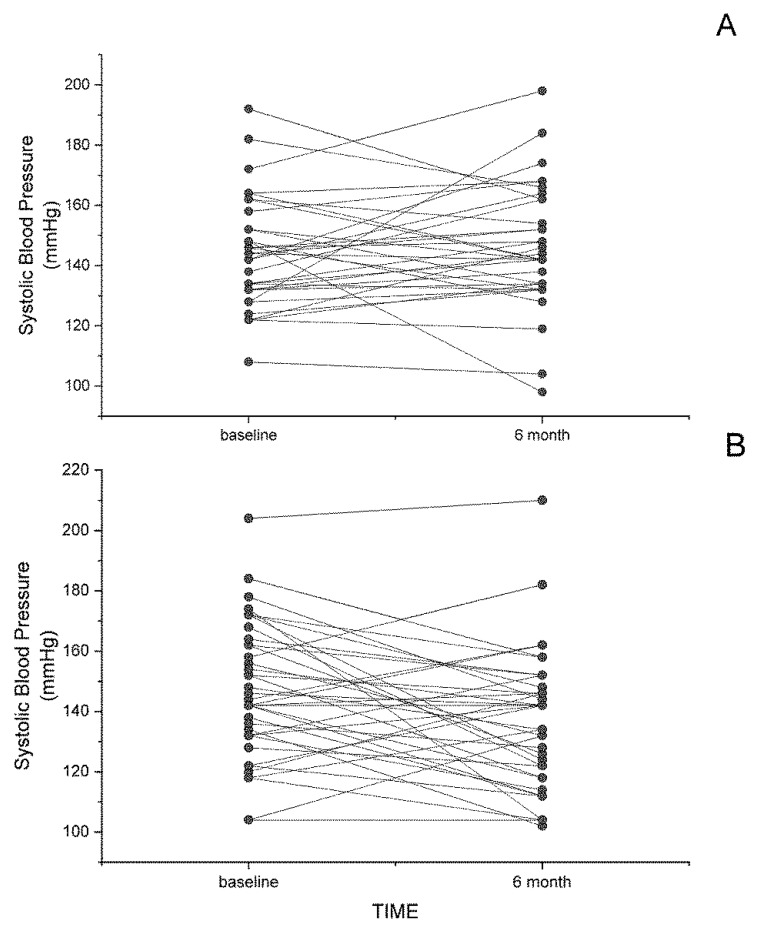
Individual values of systolic blood pressure at baseline or after 6 months of dietary supplementation with a placebo (**A**) or 30 g of milled flaxseed per day (**B**). Baseline systolic blood pressures in the placebo group were 143.5 ± 2.88 at baseline and 144.8 ± 3.33 after 6 months (*n* = 36). Baseline systolic blood pressures in the flax group were 146.6 ± 3.37 at baseline and 136.5 ± 3.53 after 6 months (*n* = 41). Results are obtained from Rodriguez-Leyva et al, 2013 [5].

In humans exhibiting symptoms of cardiovascular disease, dietary flaxseed has displayed powerfully protective effects. The most impressive involves the decrease in both systolic and diastolic blood pressure in patients with peripheral arterial disease (PAD) [5]. Both brachial and central blood pressures were significantly reduced by dietary flaxseed in this trial [5,23]. In the double-blinded, placebo-controlled, randomized FlaxPAD Trial [24], PAD patients fed 30 g of milled flaxseed every day for 6 months exhibited significant decreases in both systolic and diastolic blood pressure [5]. As shown in Figure 2, not all patients responded to the flaxseed diet with decreases in systolic blood pressure (SBP). In the placebo group, fifteen subjects showed a decrease in SBP over the 6 months, whereas twenty-one exhibited an increase in SBP. In the flax-fed group, twenty-seven subjects exhibited a decrease in SBP over the 6 months and twelve had an increase in SBP over this time. The SBP in two subjects was unchanged over this time period. The average drop in the flax group was substantial (10 mm Hg) and statistically significant. This hypotensive effect was shown as early as 1 month after commencing the flaxseed in the diet and was maintained for up to one year [5]. The decrease was sufficient to predict a 50% decrease in the incidence of myocardial infarctions and strokes [5]. This action was shown to be associated with a significant change in lipid metabolism such that the generation of oxylipins with less vasodilatory and more pro-inflammatory action was blocked [25]. This action was suggested to be due to the ALA content of the flaxseed [25]. Alternatively, in animal studies, flaxseed protein hydrolysates and flax oil decreased blood pressure in the spontaneously hypertensive rat in acute or chronic conditions, respectively [26,27]. As well, a flaxseed lignan concentrate reduced blood pressure in the deoxycorticosterone acetate (DOCA-salt) induced renal hypertensive rats [28] and in the high fat, high sugar-fed rat [29].

## 4. Dietary Flaxseed and Diabetes

Flaxseed also impacts another major disease that is growing in incidence across the globe: diabetes. Flaxseed supplementation reduced blood glucose in subjects with type 2 diabetes [30,31] and lowered blood glucose in subjects with prediabetes [32]. Flaxseed derived gum and lignan supplement also decreased blood glucose in subjects with Type 2 diabetes [33,34]. Pre-clinical studies have reported anti-hyperglycemic effects of the flaxseed lignan SDG in animals with Type I diabetes [35]. Whether SDG or flaxseed supplementation improves glycemic control in human subjects with Type 1 diabetes remains unknown and could be a topic of experimentation in the future.

## 5. Dietary Flaxseed and Cancers

Flaxseed is already used extensively in animal studies to treat a variety of cancers [36]. Perhaps the most studied cancer with respect to the impact of dietary flaxseed is breast cancer. In both experimental animal studies [36,37] and in human trials [36,37,38], dietary flaxseed has significant protective effects against breast cancer. A systematic review of 10 human trials led to the conclusion that flaxseed reduced tumour growth in women with breast cancer [36,38]. They also found evidence in support of flax-associated protection against primary breast cancer as well as a reduced risk of mortality in women living with breast cancer [38]. Beneficial effects were observed with 25 g doses of milled flaxseed [38]. 

Flaxseed lignans are nonsteroidal phytoestrogens that have a chemical structure that resembles mammalian estrogens, and hence produce estrogen-like effects in mammals. Flax lignans are metabolized by intestinal bacteria to become bioavailable in the plasma [39]. Lignans derived from food occur mainly as glucosides and undergo deglycosylation by β-glucosidases from gut microbiota before their intestinal absorption. Secoisolariciresinol diglucoside (SDG) is hydrolyzed in this way to release secoisolariciresinol (SECO). Deglycosylation of SECO is followed by demethylation by microbiota to produce dihydroxyenterodiol, which further results in the formation of enterodiol through dehydroxylation. Finally, dehydrogenation of enterodiol produces enterolactone. Enterodiol and enterolactone are absorbed from the large intestine, and ultimately appear as glucuronide and sulfate conjugates in body fluid and are excreted in urine [40]. The metabolism of SDG to enterolactone occurs in all subjects irrespective of gender [39]. A pharmacokinetic study in humans demonstrated the appearance of enterolignans in the plasma 8–10 h after ingestion of SDG, with the mean maximum plasma concentrations of enterodiol and enterolactone attaining ~14.8 h and ~19.7 h, respectively, after consumption [40]. The mean elimination half-life reported for enterodiol is ~4.4 h, and enterolactone is ~12.6 h [40]. Epidemiological studies in postmenopausal breast cancer patients have reported an association of higher blood concentrations of enterolactone with a reduced risk of breast cancer, decreased mortality rate and better survival. Clinical evidence also supports the protective effect of enterolactone in cancers of the breast, colon, prostate, gut and lung [41].

Breast cancer is not the only cancer that has shown sensitivity to dietary flaxseed or its components. Cancer of the prostate gland [42], lung [43,44], colon [45], ovary [41], endometrium [41], hepatocellular [41] and cervix have been inhibited by flaxseed.

The mechanisms responsible for the beneficial anti-oncotic action of milled flaxseed have been studied in cell culture, in animal experiments and in human trials. The rich compositions of both SDG and ALA have been postulated to play a role in the beneficial action of flaxseed on cancer [37,38,45,46]. Lignans like SDG reduce the breast cancer mortality by 33–70% and reduce all-cause mortality by 40–53% [36]. Doses of 50 mg of SDG will reduce tumours [38]. However, it is unlikely that SDG is itself responsible for the anti-tumour activity. SDG is restricted to the gastro-intestinal space and does not enter the blood stream. It is metabolized within the gastrointestinal tract to the enterolignans: enterolactone and enterodiol [47]. These compounds have estrogenic and antioxidative properties [36]. Enterolactone and enterodiol can bind to estrogen receptors and alter cell growth [37]. It is likely that these metabolites of SDG are ultimately responsible for the anti-tumour capacity of SDG [41]. The anti-inflammatory action of ALA may also play a role [41]. Flaxseed oil (enriched in ALA) also potentiates the anti-tumour action of drugs used in oncotic therapy. Flax oil administered with trastuzumab for epidermal growth factor receptor 2 positive breast cancer enhanced the anti-tumour effects of trastuzumab and significantly lowered the concentrations of trastuzumab needed to kill tumours in athymic mice [48]. Similar results have been observed using flaxseed with tamoxifen, another anti-tumour drug [37].

The cellular oncotic pathways affected by flaxseed and its bioactive components include a modulation of estrogen metabolism [36], an inhibition of cellular proliferation, angiogenesis, metastasis and inflammation, as well as a stimulation of apoptosis within the tumour [36,38,41,42,43,45,46]. The molecular targets for the anti-tumour actions of flaxseed and its bioactive components include a suppression of the phosphorylation of p-AKT, p-ERK and p-JNK kinases (resulting in a general slowing of the MAPK pathways) [43,46], inhibition of CDK4 [45], down regulation of multiple miRNAs [44], decreased expression of mRNAs for Bcl2, cell cycle proteins, ERalpha and beta and epidermal and insulin-like growth factor receptors [46].

It would appear that increasing our understanding of the value of dietary flaxseed, flax oil and SDG in this field and its efficacy in human trials may be a profitable avenue of future study. As in the case of the effects of flaxseed and its bioactive constituents in cardiovascular disease, it would appear that sufficient data already exists for the institution of these dietary therapies in specific cases of cancer prevention and treatment.

## 6. Dietary Flaxseed and the Brain

The critical role that omega-3 fatty acids obtained from marine sources play in brain development is well known [49]. This work has focused in the past primarily on the omega-3 fatty acids docosahexaenoic acid (DHA) and eicosapentaenoic acid (EPA). Whereas research has established an essential role for DHA in pre- and post-natal brain development [49,50], EPA appears to modulate behaviour and mood [50]. A loss of brain DHA content has been associated with poorer performance in spatial and learning tasks [51]. 

These data suggest that another omega-3 fatty acid, ALA, which is enriched in flaxseed, may have similar functional significance for the brain. When mothers of rats were fed flaxseed during pregnancy, the brains of newborn pups were heavier and contained significantly greater amounts of both ALA and DHA [52]. In pups given milled flaxseed or flaxseed oil soon after birth, these pups showed higher brain mass, demonstrating the value of milled flaxseed particularly in contributing to early postnatal brain development [53]. However, although dietary flaxseed may improve brain development and spatial memory, research has cautioned that the diet may depress body growth due to an imbalance between omega-3 and omega-6 fatty acid levels [54]. 

Dietary flaxseed may also improve aspects of brain function during conditions of neural disease. Maternal intake of flaxseed prevented depressive symptoms in offspring and demonstrated neuroprotection during experimental neonatal hypoxic-ischemic encephalopathy by lessening brain mass loss together with improvements in motor hyperactivity and spatial memory [55]. In mice exposed to chronic mild stress, dietary flaxseed reduced all parameters of chronic stress [56]. Supplementation to the diet with flaxseed lignans like SDG have shown anti-depressant-like effects in mice subjected to chronic stress [57] and demonstrated protective effects in cortical neurons against NMDA-induced neurotoxicity [58].

## 7. Dietary Flaxseed and Female Hormonal Status

Dietary flaxseed may also exhibit a protective effect against menopausal symptoms [59]. Several studies have examined the effects of flaxseed or its bioactive ingredients on the quality of life and the frequency and severity of hot flashes in post-menopausal women. The estrogenic action of certain metabolites of flaxseed suggested a potentially positive effect on these post-menopausal symptoms. In a study of 140 postmenopausal women, menopausal symptoms decreased and the quality of life increased in women who ingested a flaxseed supplemented diet [60]. A particularly large trial (199 women) of an unusually long duration (1 year) on a high dose of flaxseed (40 g per day) reported a significant decrease in menopausal symptoms, but this effect did not differ from the control group that ingested a wheat germ placebo [8]. Another study reported similar inconclusive evidence of a positive effect of flaxseed on menopausal symptoms with up to 90 g of flaxseed per day [61]. Further randomized controlled trials and systematic reviews of clinical trials found no significant effect of flaxseed on quality of life or hot flashes during menopause [62,63,64,65]. It is concluded that randomized, placebo-controlled trials are necessary to determine conclusive effects on menopausal symptoms. When carefully controlled trials are carried out in this manner, it would appear that flaxseed does not induce a significant effect on quality of life or the incidence and severity of hot flashes in menopausal women.

Caution has been advised for flaxseed consumption during pregnancy and lactation. SDG and flaxseed oil given to pregnant rats have shown opposite effects on total fat mass of the body. SDG administration resulted in higher fat mass, whereas flaxseed oil lowered fat mass. Furthermore, flax oil also reduced serum and milk triglyceride and cholesterol levels in female rats during lactation [66,67]. Similarly, male and female pups of mothers supplemented with flaxseed oil exhibited lower body fat mass and triglyceride levels [66,67]. With such changes in maternal and offspring biochemical parameters, it may be prudent to exercise caution when using flaxseed or its components in pregnancy. However, as no other studies have reported similar changes, randomized, placebo-controlled trials in pregnant subjects are warranted to provide any conclusive evidence.

Postmenopausal women are at an increased risk of osteoporosis. In a short 6 weeks study by Arjmandi et al [68], a slightly lower bone resorption marker (tartrate-resistant acid phosphatase) was observed in postmenopausal women receiving flaxseed. However, no influence was reported when using anabolic bone agents. Conversely, most studies have shown no effect of flaxseed consumption on bone mineral density, bone mineral content or bone turnover in postmenopausal women [69]. In an animal model, flaxseed supplementation resulted in an additional benefit when combined with estrogen therapy. Flax oil rich in ALA showed a positive effect on bone health, particularly in pathological conditions such as obesity and kidney disease. ALA may be more responsible for this improvement in osteoporotic bone conditions than the estrogenic lignan content of flaxseed [69].

## 8. Dietary Flaxseed and Skin Health

A 12 week, randomized, double-blinded study on healthy female volunteers with sensitive skin reported a positive improvement in skin properties with the ingestion of flaxseed oil. A significant decrease was noted in skin sensitivity, transepidermal water loss, skin roughness and scaling, with an increase in skin hydration and smoothness [70]. ALA was identified as the main bioactive responsible for these effects on skin and aging. Compared to a younger population, older individuals have higher concentrations of proinflammatory oxylipins including 5-HETE, 9,10,13-TriHOME and 9,12-13-TriHOME, which could explain higher levels of inflammation in this older demographic [71]. Dietary supplementation of flaxseed has been shown to correct the balance of pro- and anti-inflammatory oxylipins and thus may exert a healthy effect on aging [71]. 

## 9. Dietary Flaxseed and Gastro-Intestinal Health

The influence of the microbiome on human and animal health is receiving increasing attention from the research community [72]. However, our understanding of the impact of dietary flaxseed on the gut microbiome in either healthy or diseased populations is limited. It was known that the lignans that are unusually high in content within flaxseed need to be metabolized by intestinal bacteria in order to gain access to the systemic circulation in humans. The production of enterolignans like enterodiol and enterolactone from SDG is best generated from milled flaxseed and defatted flaxseed meal, in comparison to a variety of other foods that contain SDG [73]. This intestinal metabolism is accomplished via the biotransformative action of specific intestinal bacteria [74] including *Ruminococcus bromii* and *Ruminococcus lactaris* [75]. Others have found *Lactobacillus casei* and *Lactobacillus acidophilus* were important for the digestion of whole flaxseed in order to increase enterodiol bioaccessibility [76].

Early studies have shown the capacity of flaxseed supplementation in the diet to alter the bacterial flora in the intestines of animals. For example, flaxseed supplemented to the diet of mice will alter *Enterobacteriaceae* diversity and prevalence in the feces and cecum [77]. Power and colleagues [78] showed that a flaxseed supplemented diet will alter the microbial microenvironment in the colon with a 20-fold increase in *Prevotella* spp. and a 30-fold decrease in *Akkermansia muciniphila* abundance. Others have shown that a combined high fat, flaxseed and fish oil supplemented diet increased the abundance of intestinal *Bifidobacterium* [79].

**Figure 3:**
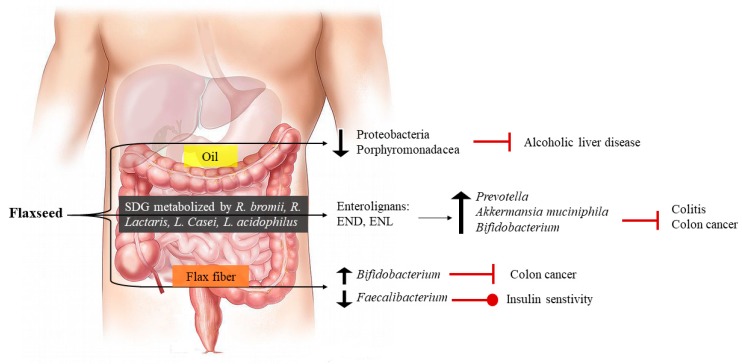
The effect of dietary flaxseed on the gastrointestinal health.

Changes in specific bacteria in the microbiome may have implications on disease progression (Figure 3). Dietary flax oil consumption can, under some conditions, reduce the presence of *Proteobacteria* and *Porphyromonadaceae* in the gut microbiome, which may have a positive impact on alcoholic liver disease [80]. Defatted flaxseed meal (which is a partially delipidated form of flaxseed) reduced the appearance of aberrant crypt foci in the colon of mice through an increase in the expression of *Bifidobacterium* [81]. Daily intake of flaxseed mucilage resulted in changes in the abundance of 33 different metagenomics species in the gut microbiota, including eight different species of *Faecalibacterium*. This altered insulin sensitivity in these women [82]. These data contrast with those of Lagkouvardos et al [75], who found that flaxseed did not significantly change the fecal microbiome nor the abundance of the dominant bacterial species present. Others have also challenged the efficacy of dietary flaxseed to alter the appearance of disease based upon a change in the gut microbiome. For example, a 5% flaxseed supplemented diet had no protective effect against the development of intestinal tumours [83]. Caution in the use of flaxseed in the diet has also been introduced by the results of Maatanen and co-workers, who showed that whereas a reduced fat diet could protect against colitis, the high levels of PUFAs provided by flaxseed supplementation to the diet diminished these beneficial effects [84]. 

The potential for dietary flaxseed to replace or reduce the use of constipation medication in different populations at risk (the elderly, the ill, in personal health care homes, etc.) and the reduction in side-effects associated with conventional constipation medication is worthy of study. Two recent randomized trials have generated results of great interest for the introduction of flaxseed into diets for relief from constipation. In the first trial [30], flaxseed baked into cookies was ingested in constipated patients with Type 2 diabetes. They found the flaxseed reduced constipation symptoms, weight, fasting plasma glucose, triglycerides and LDL and HDL cholesterol levels. In a subsequent trial using the same type of patient population, flaxseed affected all of these parameters once again and, importantly, was superior in its capacity to reduce constipation symptoms to psyllium [85]. Flaxseed may also be of use in reducing the symptoms of irritable bowel syndrome; however, further research is required [86]. Flaxseed oil was also beneficial in reducing experimental diarrhea [87].

The mechanism of action of flaxseed in stimulating fecal output during constipation clearly lies largely with its high fiber content. It is not surprising, therefore, that even partially defatted flaxseed meal was a valuable laxative in both normal and constipated condition [78]. However, flaxseed oil has been shown to act on intestinal muscarinic receptors and blocks K+ channels [77], actions that would both lead to modifications of intestinal motility.

## 10. Toxicity of Flaxseed

Although no toxicity has ever been reported in clinical studies with dietary supplementation of flaxseed, some compounds within flaxseed such as cyanogenic glycosides and linatine have been identified as potential toxic compounds. Cyanogenic glycosides such as linamarin, linustatin, neolinustatin, lotaustralin and amygdalin are nitrogenous secondary plant metabolites [3]. These compounds are not exclusively found in flaxseed, but are also present in other food items including apples, spinach and cassavas [88,89,90]. The glycoside is converted by intestinal β-glycosidase to cyanohydrin, which then decomposes to hydrogen cyanide [90]. In flaxseed, this process is catalyzed by two distinct enzymes: linustatinase and linamarase β-glucosidase [90]. Hydrogen cyanide could cause acute cyanide poisoning, which may place the respiratory and nervous system at risk [3]. However, no increase in plasma cyanide levels above baseline have been observed with the consumption of 15–100 g of flaxseed [90]. The linustatin and neolinustatin found in flaxseed are thought to be the lowest cyanide producers compared to other cyanogenic glycosides. This is because flaxseed glycosides have a gentiobiose moiety that needs to be hydrolyzed to glucose. The cyanohydrin formed by metabolism is highly stable [90], which, therefore, resists any spontaneous decomposition to hydrogen cyanide. Theoretically, 1–2 tablespoons of flaxseed will produce approximately 5–10 mg of hydrogen cyanide after ingestion. This is highly unlikely to cause toxicity for three reasons: a) 50–60 mg dose of cyanide is required to cause acute toxicity; b) the human body can routinely detoxify up to 100 mg/day of cyanide [3,89] and c) cooking destroys cyanide (cyanide is heat labile [91]). Human studies with 50 g/day flaxseed did not increase urinary thiocyanate levels [3]. Based on these data, humans would need to consume the unrealistic amount of 1 kg of flaxseed daily for cyanide toxicity to ever manifest itself. 

Another potentially toxic compound is linatine (antipyridoxin factor), which has been identified as a vitamin B6 antagonist in chicks [89]. However, flaxseed has never been shown to induce vitamin B6 deficiency in clinical studies [91]. Other compounds such as phytic acid and trypsin inhibitor have also been suggested to induce negative effects on the nutritional status after flaxseed ingestion. However, once again, no studies have reported any alterations in zinc status due to phytic acid, or any difference in trypsin inhibitor activity in flaxseed compared with canola or soybean seeds [89,91]. In conclusion, it is important to recognize that no definitive scientific data have been produced to support the concept of toxicity from dietary flaxseed because of any of these compounds.

## 11. Conclusions

Supplementation of the diet with milled flaxseed has many healthy benefits to the body. Although cardiovascular disease and canceer are probably the best researched areas that have shown convincing evidence of a beneficial action for dietary flaxseed, other areas like gastro-intestinal health and diabetes have also been receptive to the beneficial effects of dietary flaxseed. Other areas in human health require further research to make definitive conclusions but the preliminary data is encouraging. With little or no evidence of toxicity for dietary supplementation with flaxseed, there appears to be a clear argument to support its inclusion in the daily diet and little reason to oppose it.
